# Efficacy and safety of subanesthetic doses of esketamine combined with propofol in painless gastrointestinal endoscopy: a prospective, double-blind, randomized controlled trial

**DOI:** 10.1186/s12876-022-02467-8

**Published:** 2022-08-20

**Authors:** Yongtong Zhan, Shuqing Liang, Zecheng Yang, Qichen Luo, Shuai Li, Jiamin Li, Zhaojia Liang, Yalan Li

**Affiliations:** 1 Department of Anesthesiology, The First Affiliated Hospital of Jinan University, Guangzhou, 510630 China; 2 Department of General Surgery, The First Affiliated Hospital of Jinan University, Guangzhou, China

**Keywords:** Esketamine, Propofol, Gastrointestinal endoscopy, Randomized controlled trial

## Abstract

**Background:**

Painless gastrointestinal endoscopy is widely used for the diagnosis and treatment of digestive diseases. At present, propofol is commonly used to perform painless gastrointestinal endoscopy, but the high dose of propofol often leads to a higher incidence of cardiovascular and respiratory complications. Studies have shown that the application of propofol combined with ketamine in painless gastrointestinal endoscopy is beneficial to reduce the dosage of propofol and the incidence of related complications. Esketamine is dextrorotatory structure of ketamine with a twice as great anesthetic effect as normal ketamine but fewer side effects. We hypothesized that esketamine may reduce the consumption of propofol and to investigate the safety of coadministration during gastrointestinal endoscopy.

**Methods:**

A total of 260 patients undergoing painless gastrointestinal endoscopy (gastroscope and colonoscopy) were randomly divided into P group (propofol + saline), PK1 group (propofol + esketamine 0.05 mg/kg), PK2 group (propofol + esketamine 0.1 mg/kg), and PK3 group (propofol + esketamine 0.2 mg/kg). Anesthesia was achieved by 1.5 mg/kg propofol with different doses of esketamine. Propofol consumption per minute was recorded. Hemodynamic index, pulse oxygen saturation, operative time, induction time, awakening status, orientation recovery time, adverse events, and Mini-Mental State Examination (MMSE) were also recorded during gastrointestinal endoscopy.

**Results:**

Propofol consumption per minute was 11.78, 10.56, 10.14, and 9.57 (mg/min) in groups P, PK1, PK2, and PK3, respectively; compared with group P, groups PK2 and PK3 showed a decrease of 13.92% (*P* = 0.021) and 18.76% (*P* = 0.000), respectively. In all four groups, systolic blood pressure (SBP), diastolic blood pressure (DBP), heart rate (HR), but not pulse oxygen saturation (SpO_2_) significantly decreased (*P* = 0.000) immediately after administration of induction, but there were no significant differences between the groups. The induction time of groups P, PK1, PK2, and PK3 was 68.52 ± 18.394, 64.83 ± 13.543, 62.23 ± 15.197, and 61.35 ± 14.470 s, respectively (*P* = 0.041). Adverse events and psychotomimetic effects were observed but without significant differences between the groups.

**Conclusions:**

The combination of 0.2 mg/kg esketamine and propofol was effective and safe in painless gastrointestinal endoscopy as evidenced by less propofol consumption per minute, shorter induction time, and lower incidence of cough and body movement relative to propofol alone. The lack of significant differences in hemodynamic results, anesthesia-related indices, adverse events, and MMSE results showed the safety to apply this combination for painless gastrointestinal endoscopy.

*Trial registration* This study was registered with China Clinical Trial Registration on 07/11/2020 (registration website: chictr.org.cn; registration numbers: ChiCTR https://clinicaltrials.gov/ct2/show/2000039750).

## Background

Gastrointestinal (GI) endoscopy is the gold standard for the diagnosis of digestive tract diseases and is also widely used for the treatment of digestive tract diseases [1]. Almost half of the individuals underwent GI endoscopy without sedation reported pain or discomfort [2–4]. Poor pain management and patient’s anxiety can decrease procedure adherence[5].The introduction of sedative agents during GI endoscopy help to relief anxiety and pain. Painless GI endoscopy is much more popular with the administration of sedative agents, with more than 98% of GI endoscopies performed with sedation in the United States [6]. As the painless GI endoscopy has become popular among patients, it has also brought new challenges to anesthesiologists to deal with anesthesia complications such as hypotension, respiratory depression, nausea, vomiting, and even death.

When facing all these challenges, anesthesiologists must make an anesthesia plan before the GI procedure, ensuring that patients are stable and comfortable during the procedure, wake up quickly, and leave the hospital safely as soon as possible [7]. In clinical practice, propofol is one of the most commonly used drugs for painless GI endoscopy [8]. However, given that propofol does not have analgesic effects, large doses are needed to meet the requirements of painless GI endoscopy, which increases the risk of dose-related complications such as hypotension and hypoxemia [9, 10]. Therefore, it is appropriate to apply propofol in combination with other anesthetics, such as opioids (e.g., remifentanil, sufentanil), midazolam, etomidate, and ketamine, to reduce the consumption of propofol and the related complications. To date, there is no agreement on the optimal sedation regimen to facilitate GI endoscopy in an effective, safe, and satisfying manner for both patients and endoscopists [11].

Esketamine, an antagonist of the N-methyl-D-aspartic acid receptor, has analgesic and anesthetic effects and causes less cardiorespiratory and respiratory depression. It also leads to fewer psychotomimetic effects and less secretion [12, 13]. Therefore, it would be useful to examine whether there is any advantage in esketamine usage in painless GI endoscopy. We conducted a double-blinded, randomized controlled study to determine the safety and efficacy of subanesthetic doses of esketamine combined with propofol in painless GI endoscopy. We hypothesized that esketamine may reduce the consumption of propofol and that it is safe to coadminister propofol and esketamine during GI endoscopy.

## Methods

### Ethics and trial registration

China Ethics Committee of Registering Clinical Trials approved the protocol (Ethics Number: CHIECRCT20210050), and the study was registered in the Chinese Clinical Trial Registry (ChiCTR 2000039750; 07/11/2020). All participants signed the informed consent. The full trial protocol can be accessed at the following website: chictr.org.cn.

### Inclusion and exclusion criteria

A prospective, double-blind, randomized controlled study was conducted at the First Affiliated Hospital of Jinan University.Between November 9, 2020, and September 1, 2021, patients scheduled for elective painless GI endoscopy were eligible for participation in this trial if they: (i) were 18–60 years old; (ii) had American Society of Anesthesiologists Physical Status (ASA PS) I to II; (iii) had a full understanding of the purpose and significance of this trial; and (iv) were able to sign an informed consent form.

Exclusion criteria were as follows: (i) a history of unregulated diabetes, hypertension, and hypotension; (ii) myocardial infarction within 6 months or unstable angina pectoris; (iii) III degree atrioventricular block; (iv) severe snoring; (v) sleep apnea syndrome; (vi) decompensated liver function or renal function; (vii) dialysis treatment; (viii) psychosocial disease or cognitive dysfunction; (ix) a history of psychotropic drugs and narcotic drug abuse; (x) allergy to or contraindication for the drugs used in this study; (xi) participation in clinical trials of other drugs within 3 months; (xii) other circumstances in which the investigator determined that a patient was not suitable for participation in the clinical trial.

### Sample size estimation

According to our pre-experiment, the mean value and standard deviation of propofol consumption per minute in each group were obtained, and the inspection efficiency was set at 0.90, while the inspection level was set at 0.05. A required sample size of 62 subjects per group was calculated using PASS 11 software (NCSS, Kaysville, Utah). Considering a loss rate of 5%, a total of 260 subjects (65 subjects per group) were required.

### Randomization and blindness

The random sequence generator of STATA MP 14 software generated 260 random numbers and divided them into four groups. The patients were assigned into four groups: group P (propofol); group PK1 (propofol + eskatemine 0.05 mg/kg); group PK2 (propofol + eskatemine 0.10 mg/kg); and group PK3 (propofol + eskatemine 0.20 mg/kg). For each patient enrolled, a researcher drew an envelope to determine the patient’s grouping, but the patient was not informed of the grouping. Except for propofol, clinical researchers were given syringes with clear solutions in the same bottles with codes according to the randomization order. The researchers who performed the randomization and blinding procedure did not participate in the follow-up study. Other investigators were not informed of the grouping of the study and the experimental drugs. To ensure allocation concealment, randomization results were sealed until the end of the study.

### Anesthesia sedation process

After routine GI endoscopy preparation and fasting for 6 h before the procedure, the patients arrived at the endoscopy center and were asked about basic information. If the patients met the inclusion criteria, they signed an informed consent form. Grouping was determined according to the randomization. Before sedation, the study drugs were diluted with normal saline in a 10 mL syringe by a blindless nurse, with final concentrations of 0.5 mg/mL, 1 mg/mL, and 2 mg/mL for esketamine.

**Fig. 1:**
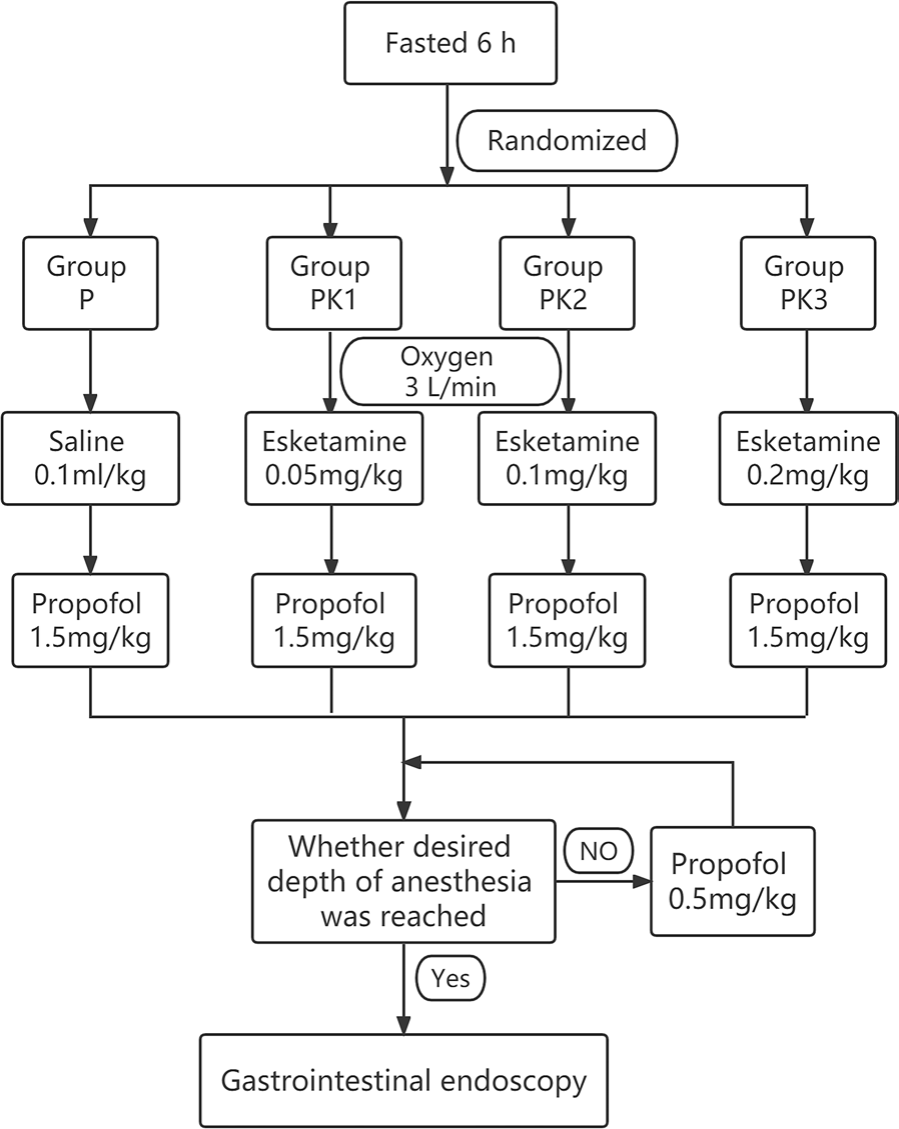
Flow chart of anesthesia operation

The procedure of sedation is shown in Fig. 1. In our study, all participants underwent gastroscopy first and tnen colonoscopy after anesthesia induction. They were sedative through the whole procedure (gastroscopy and colonoscopy). A 20 G IV cannula was inserted via the antecubital vein for fluid infusion. Standard monitoring included blood pressure (BP), heart rate (HR), and pulse oximetry (SPO_2_) at T_0_, followed by Mini-Mental State Examination (MMSE) [14]. Data collection and MMSE was performed by a blindness anesthesiologist who didn't take part in the administration of drugs.

Oxygen supplementation of 3 L/min was delivered via a nasopharynx tube throughout the study. BP, HR, and SPO_2_ at T_1_ were recorded 5 min before anesthesia induction. The patient was sedated by the same blindless anesthesiologist using the prepared test drug and propofol. The same dose of normal saline (0.1 mL/kg), eskatemine (0.05 mg/kg, 0.10 mg/kg, and 0.20 mg/kg) for the P group, PK1 group, PK2 group, and PK3 group, respectively, were injected slowly (more than 6 s) followed by 1.5 mg/kg propofol for anesthesia induction. BP, HR, and SPO_2_ at T_2_ were recorded immediately after administration of induction. When the patient’s eyelash reflex disappeared and there was no significant movement after the gastroscope introduction, the same doctor at the endoscopy center began to perform the endoscopy. BP, HR, and SPO_2_ at T_3_ were recorded immediately after gastroscope insertion. A rescue dose of propofol (0.5 mg/kg) was used if body movement, cough, or swallowing occurred during the procedure. BP, HR, and SPO_2_ were recorded immediately before (T_4_) and after (T_5_) colonoscopy. After the GI endoscopy examination, the patient was roused every 2 min. If the patient was able to open their eyes and nod their head, the time of awakening was recorded. Accordingly, 10 min after the patient awakened, MMSE was evaluated by the same anesthesiologist.

During the operation process, SpO_2_ < 95% was corrected by chin lifting, while SpO_2_ < 90% was corrected by face mask ventilation. Phenylephrine 0.1 mg was injected when BP was below 30% of the baseline level, and atropine 0.25 mg was injected when HR was below 50/min. Other respiratory and cardiovascular adverse events were recorded and managed in accordance with the clinical operation standard. Before leaving the operating room, BP, HR, and SPO_2_ (T_6_) as well as the ambulation time were recorded.

### Primary and secondary outcome

The primary outcome of this study was propofol consumption per minute (mg/min). The total consumption of propofol and the duration of the procedure were recorded and then propofol consumption per minute was calculated. Secondary outcomes included hemodynamic index (T_0_–T_6_), induction time (from the injection of the study medication to the disappearance of eyelash reflex), procedure time (from the insertion of the gastroscope to the withdrawal of the colonoscope), delayed awakening (awakening time more than 6 min is defined as delayed awakening), orientation recovery time (consciousness return to normal walking), MMSE results, and adverse events. Adverse events included propofol injection pain, hypoxemia (SPO_2_ < 95%), hypotension (blood pressure < 30% of the basal blood pressure or systolic blood pressure), hypertension (blood pressure > 30% of the basal blood pressure), arrhythmia, nausea and vomiting, dizziness, cough, body movement, apnea, excessive oral secretion, and psychotomimetic effects.

### Statistical analysis

PASS 11 software was used to calculate the sample size. GraphPad Prism version 8.3.0 (San Diego, California) was used for statistical analysis. Continuous variables were shown as mean ± SD, and categorical variables were expressed as N (%) of patients. All data were checked for normal distribution using the Kolmogorov–Smirnov test and histograms. For continuous variables, ANOVA followed by Student–Newman–Keuls test was performed. For discrete variables, Kruskal–Wallis *H* rank sum test followed by Tamhane’s T2 method was used. Repeated-measurement data were analyzed by ANOVA with a repeated-measurement design. For categorical variables, the pearson chi-square test or fisher's exact test was applied. *P* values < 0.05 were considered statistically significant.

## Results

### Patient inclusion and characteristics

**Fig. 2:**
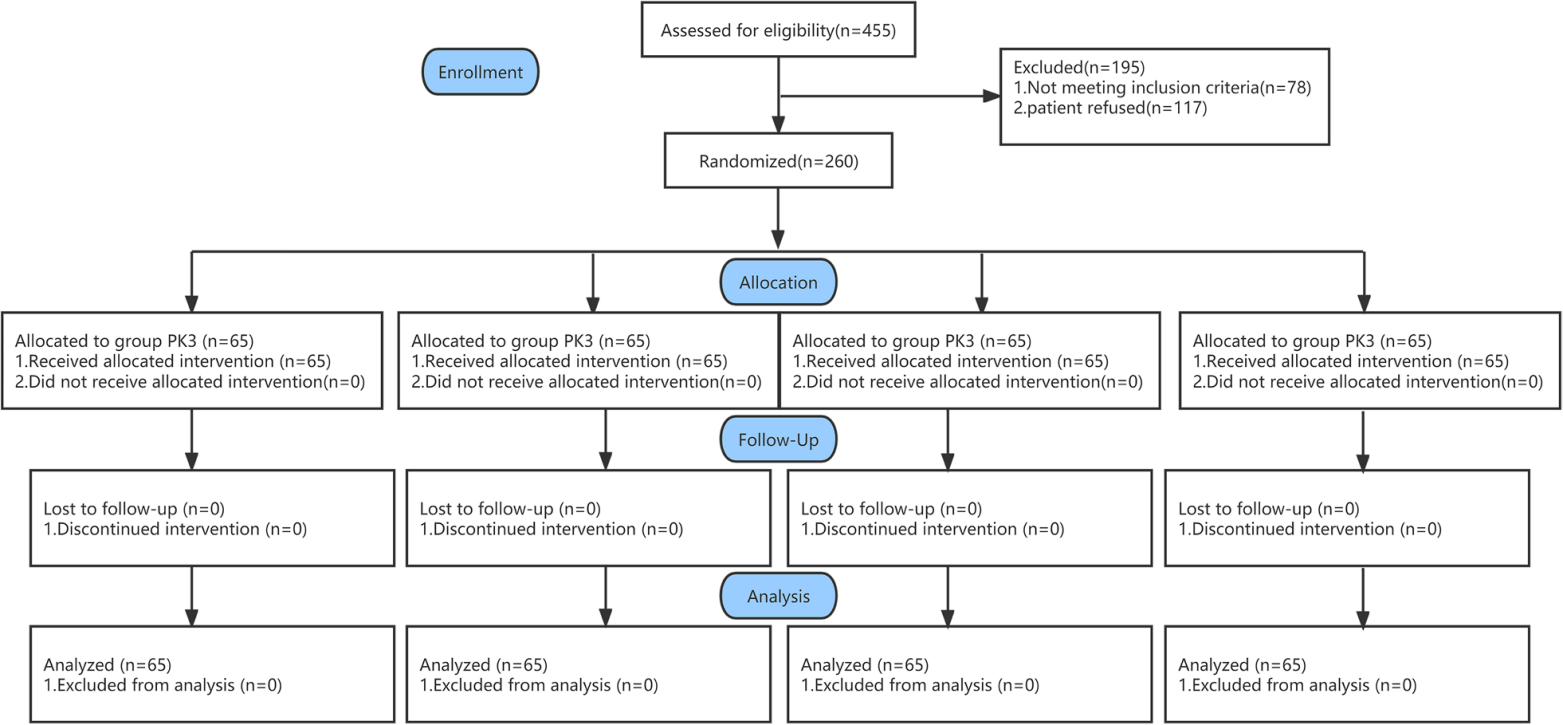
Consort flow diagram

Figure 2 shows the study flow diagram. There were 260 patients meeting the inclusion criteria and they were randomized into the study groups. The patients’ demographic characteristics and baseline values are shown in Table 1. There were no significant differences in the patients' characteristics, comorbidities, and baseline values among the four groups.

**Table 1:** Patients’ demographic characteristics

	Group P	Group PK1	Group PK2	Group PK3	*P* value
Age (years)	44.94 ± 10.031	42.71 ± 10.148	45.89 ± 9.292	44.38 ± 10.233	0.785
Gender (F)	38 (58.5%)	38 (58.5%)	32 (49.2%)	30 (46.2%)	0.369
Weight (Kg)	61.32 ± 10.053	60.77 ± 9.465	62.97 ± 9.352	60.52 ± 9.816	0.918
Height (cm)	164.20 ± 7.688	163.28 ± 7.966	165.12 ± 7.962	165.65 ± 7.787	0.969
BMI	22.67 ± 2.755	22.74 ± 2.664	23.06 ± 2.770	21.99 ± 2.730	0.961
ASA (I)	43 (66.2%)	39 (60.0%)	42 (64.6%)	42 (64.6%)	0.896
Gastroscope	65(100%)	65(100%)	65(100%)	65(100%)	
Colonoscopy	65(100%)	65(100%)	65(100%)	65(100%)	
Comorbidities					
Hypertension	19 (29.2%)	21 (32.3%)	19 (29.2%)	16 (24.6%)	0.812
Diabetes	6 (9.2%)	2 (3.1%)	6 (9.2%)	3 (4.6%)	0.377
Asthma	0 (0%)	0 (0%)	1 (1.5%)	0 (0%)	1.000
COPD	0 (0%)	0 (0%)	0 (0%)	0 (0%)	1.000
Tachycardia	7 (10.8%)	2 (3.1%)	8 (12.3%)	4 (6.2%)	0.194
IHD	0 (0%)	1 (1.5%)	0 (0%)	0 (0%)	1.000
Baseline					
SpO_2_ (%)	98.00 ± 0.919	97.82 ± 1.059	98.03 ± 1.000	98.05 ± 0.909	0.504
SBP (mm Hg)	128.32 ± 17.057	126.75 ± 17.317	128.11 ± 17.540	124.85 ± 15.294	0.624
DBP (mm Hg)	78.91 ± 10.728	78.57 ± 11.727	80.25 ± 9.548	75.46 ± 10.499	0.073
HR (bpm)	85.46 ± 16.260	82.86 ± 14.992	84.43 ± 12.993	84.35 ± 14.303	0.793

Data are presented as mean ± SD, or number (%).

*BMI* body mass index, *ASA* American Society of Anesthesiologists, *COPD* chronic obstructive pulmonary disease, *IHD* ischemic heart disease, *DBP* diastolic blood pressure, *SBP* systolic blood pressure, *HR* heart rate

### Propofol consumption per minute

**Fig. 3:**
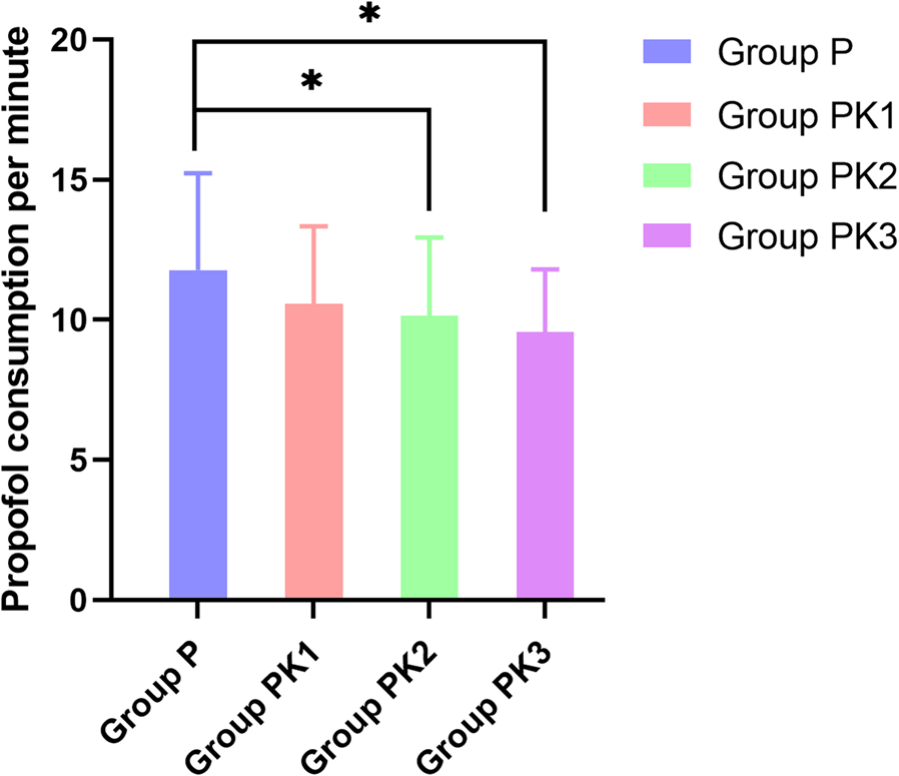
Propofol consumption per minute. Patients were treated with propofol alone or propofol with different doses of esketamine. Propofol consumption per minute was recorded at the end of the gastrointestinal endoscopy. N = 65, *P < 0.05

Propofol consumption per minute was 11.78, 10.56, 10.14, and 9.57 (mg/min) in groups P, PK1, PK2, and PK3, respectively (Fig. 3). Compared with group P, propofol consumption per minute in groups PK2 and PK3 decreased by 13.92% (*P* = 0.021) and 18.76% (*P* = 0.000), respectively. There were no significant differences in propofol consumption per minute among the three esketamine groups.

### Hemodynamic results

**Fig. 4:**
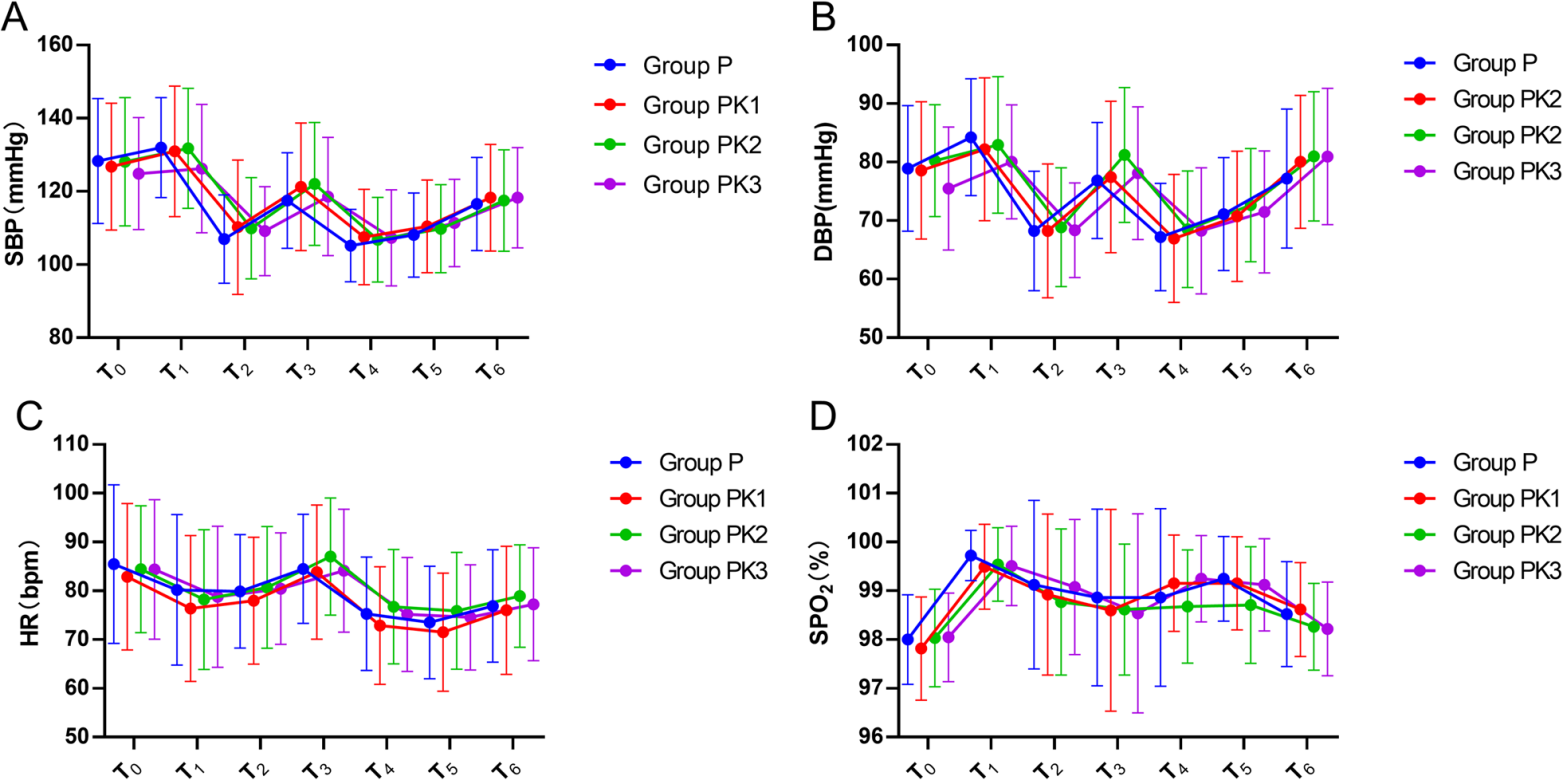
The results of repeated measurements of hemodynamic parameters. Systolic blood pressure (**A**), diastolic blood pressure (**B**), heart rate (**C**), and pulse oximetry (**D**) of patients treated with propofol alone or in combination with different doses of esketamine during gastrointestinal endoscopy

SBP, DBP, HR, but not SpO_2_ significantly decreased (*P* = 0.000) at T2, the time immediately after administration of induction. SBP, DBP, HR, and SpO_2_ were significantly higher at T6 (*P* > 0.05), the time before patients left the operating room, compared with the baseline value (Fig. 4). The area under the curve(AUC) of SBP (*P* = 0.097), DBP (*P* = 0.253), HR (*P* = 0.838), and SpO_2_ (*P* = 0.087) did not differ among the groups (Table 2). In general, there were no significant differences in hemodynamic parameters between the groups.

**Table 2:** The AUC of SBP, DBP, HR, and SpO_2_

	Group P	Group PK1	Group PK2	Group PK3	*P* value
AUC (T_0_–T_6_) of SBP	97.10 ± 57.372	84.85 ± 46.859	93.26 ± 44.072	77.83 ± 40.767	0.097
AUC (T_0_–T_6_) of DBP	51.12 ± 20.910	51.39 ± 26.653	47.71 ± 19.993	44.49 ± 21.689	0.253
AUC (T_0_–T_6_) of HR	52.82 ± 42.436	55.76 ± 35.907	49.92 ± 28.269	52.87 ± 36.547	0.838
AUC (T_0_–T_6_) of SpO_2_	8.51 ± 4.411	8.52 ± 4.842	6.81 ± 3.887	7.70 ± 4.406	0.087

Data are presented as mean ± SD.

*AUC* the area under the curve, *DBP* diastolic blood pressure, *SBP* systolic blood pressure, *HR* heart rate

### Evaluation of anesthesia-related indices

Induction times of group P, PK1, PK2, and PK3 were 68.52 ± 18.394, 64.83 ± 13.543, 62.23 ± 15.197, and 61.35 ± 14.470 s, respectively (*P* = 0.041). The induction time of group PK3 was 7.17 s shorter than that of group P. There were 8, 9, 8, and 5 patients who experienced an awakening delay in groups P, PK1, PK2, and PK3, respectively (*P* = 0.716). There were no significant differences in procedure time, orientation recovery time, and awakening status between the groups (Table 3).

**Table 3:** Anesthesia-related indices

	Group P	Group PK1	Group PK2	Group PK3	*P* value
Induction time (s)	68.52 ± 18.394	64.83 ± 13.543	62.23 ± 15.197	61.35 ± 14.470※	0.041
Procedure time (min)	20.54 ± 6.917	19.31 ± 4.603	21.23 ± 6.675	20.51 ± 5.420	0.325
Orientation recovery time (min)	14.26 ± 3.989	14.22 ± 4.615	14.17 ± 3.547	15.02 ± 3.851	0.584
Awakening status (D)	8 (12.3%)	9 (13.8%)	8 (12.3%)	5 (7.7%)	0.716

Data are presented as mean ± SD or n (%); D, delayed; ※*P* < 0.05 compared with group P

### Incidence of adverse events

We did not find any significant clinical complications during the study. However, the recorded adverse events included injection pain, hypoxemia, hypotension, cough, body movement, dizziness, excessive oral secretion (two patients in group PK3), hypertension (one patient in group PK1), and psychotomimetic effects (Tables 4 and 5). Compared with group P, group PK3 showed less cough (*P* = 0.048) and body movement (*P* = 0.004). One patient in group PK1 developed an unintentional tremor, which gradually disappeared after half an hour of rest. One patient in group P and one in group PK3 had unintentional lacrimation without depression or sadness. One patient in group PK3 was agitated, reporting a very pleasant mood and involuntarily wanting to laugh. One patient in group PK3 had nightmares and felt tormented. No arrhythmia, nausea and vomiting, apnea (Table 4), hallucinations, delirium, depression, and other Psychoactive effects were observed (Table 5).

**Table 4:** Adverse events

	Group P	Group PK1	Group PK2	Group PK3	*P* value
Injection pain	3 (4.6%)	7 (10.8%)	2 (3.1%)	1 (1.5%)	0.149
Hypoxemia	14 (21.5%)	16 (24.6%)	23 (35.4%)	16 (24.6%)	0.297
Hypotension	11 (16.9%)	8 (12.3%)	3 (4.6%)	4 (6.2%)	0.072
Cough	46 (70.8%)	34 (52.3%)	37 (56.9%)	31 (47.7%)※	0.048
Body movement	54 (83.1%)	55 (84.6%)	42 (64.6%)	39 (60.0%)※†	0.001
Dizziness	7 (10.8%)	9 (13.8%)	6 (9.2%)	11 (16.9%)	0.563
Excessive oral secretion	0 (0%)	0 (0%)	0 (0%)	2 (3.1%)	0.247
Hypertension	0 (0%)	1 (1.5%)	0 (0%)	0 (0%)	1.000
Arrhythmia	0 (0%)	0 (0%)	0 (0%)	0 (0%)	1.000
Nausea and vomiting	0 (0%)	0 (0%)	0 (0%)	0 (0%)	1.000
Apnea	0 (0%)	0 (0%)	0 (0%)	0 (0%)	1.000

Data are presented as number (%); ※*P* < 0.05 compared with group P, †*P* < 0.05 compared with group PK1

**Table 5:** Psychoactive effects

	Group P	Group PK1	Group PK2	Group PK3	*P* value
Tremor	0 (0%)	1 (1.5%)	0 (0%)	0 (0%)	1.000
Lacrimation	1 (1.5%)	0 (0%)	0 (0%)	1 (1.5%)	1.000
Agitation	0 (0%)	0 (0%)	0 (0%)	1 (1.5%)	1.000
Nightmare	0 (0%)	0 (0%)	0 (0%)	1 (1.5%)	1.000
Hallucination	0 (0%)	0 (0%)	0 (0%)	0 (0%)	1.000
Delirium	0 (0%)	0 (0%)	0 (0%)	0 (0%)	1.000
Tristimania	0 (0%)	0 (0%)	0 (0%)	0 (0%)	1.000

Data are presented as numbers (%)

### MMSE results

We performed MMSE before sedation and 10 min after the patient’s awakening. There were no significant changes in MMSE scores after sedation (*P* = 0.210) in any of the groups. There were also no significant differences among the four groups (*P* = 0.551, Table 6).

**Table 6:** MMSE results

	Group P	Group PK1	Group PK2	Group PK3	*P* value
MMSE (before)	27.22 ± 2.661	27.66 ± 2.340	27.92 ± 2.189	28.32 ± 1.880	0.551
MMSE (after)	28.06 ± 2.480	27.82 ± 2.164	28.03 ± 2.143	28.20 ± 2.009	0.551

Data are presented as mean ± SD; MMSE: Mini-Mental State Examination

## Discussion

The increased use of the painless GI endoscopy has led to an increase in sedation practices for patients [6, 9], and this study aimed to evaluate the efficacy and safety of esketamine–propofol combination compared to propofol alone in GI endoscopy. We found that the use of 0.1 mg/kg and 0.2 mg/kg esketamine reduced the need for propofol by 13.92% and 18.76%, respectively. The combination also shortened the induction time and reduced the incidence of cough and body movement during GI endoscopy. The results of this study indicated a similar performance of the combination esketamine–porpofol and propofol alone in terms of hemodynamic stability and adverse events.

In this study, subanesthetic doses of esketamine combined with propofol showed a significant reduction in propofol consumption per minute compared with propofol alone during the GI endoscopy. This result showed the efficacy of the subanesthetic dose of esketamine–propofol combination during GI endoscopy sedation. Yang et al. [15] also reported the effectiveness of esketamine as an adjunct to propofol target-controlled infusion for gastrointestinal endoscopy in elderly patients. Eberl and colleagues found that this combination was also effective in Endoscopic retrograde cholangiopancreatography (ERCP). They used low-dose (0.15 mg/kg) esketamine combined with propofol in patients undergoing ERCP and found that low-dose esketamine significantly reduced propofol consumption [16]. Since esketamine has both sedative and analgesic effects, esketamine combined with propofol deepens the level of sedation, causing a reduction in rescue doses of propofol during GI endoscopy and finally leading to the reduction of propofol consumption.

Importantly, esketamine is a very new medication being used clinically; thus, it is necessary to evaluate its safety profiles during GI endoscopy sedation. We recorded several elements during the procedure, which included hemodynamic characteristics, induction time of anesthesia, awakening and recovery status, cough, body movement, adverse events, and cognitive function.

We did not find any significant difference among the four groups in terms of hemodynamic characteristics, including HR, SBP, and DBP, at different time points. However, SBP, DBP, and HR decreased immediately after administration of induction and increased to the baseline value at the time before patients left the operating room in all four groups. In contrast, another study showed that the combination of esketamine and propofol caused more stable hemodynamics [15]. The doses of esketamine in that study were 0.25 mg/kg and 0.5 mg/kg, which are relatively higher than the concentrations in our study. With sympathetic excitatory effects of esketamine, the hemodynamics of group PK would theoretically be more stable than that of group P [17]. However, the dose of esketamine used in our study was low and the sympathetic excitatory effect of esketamine may be offset by the cardiovascular depression of relatively high doses of propofol during the procedure.

Induction with 0.2 mg/kg esketamine combined with propofol shortened the induction time of anesthesia in our study. However, another study showed no difference in the induction time between ketamine combined with propofol and propofol alone when applied to painless GI endoscopy in elderly people [11]. The higher potency of esketamine compared with ketamine may be a possible reason for the shorter induction time. As for awakening and recovery status, our study and other studies [11, 16] found no differences between esketamine–propofol combination and propofol alone, suggesting that esketamine may not affect the awakening of patients sedated with propofol. Additionally, in a study of esketamine compared with ketamine applied to gastroscopy, it was found that the recovery time and orientation recovery time were shorter in patients with esketamine, suggesting that esketamine is more suitable for gastroscopy than ketamine [12]. Avoiding cough and body movement is of great importance for improving the comfort of patients and quality of endoscopy. In this study, we found that the group treated with 0.2 mg/kg esketamine combined with propofol had a lower incidence of cough and body movement. Yin et al. [11] also found that the incidence of cough and body movement was lower when elderly patients were sedated with ketamine combined with propofol during GI endoscopy.

We also evaluated several types of adverse events to estimate the safety of esketamine sedation, including hypotension, hypoxemia, propofol injection pain, dizziness, excessive oral secretion, hypertension, arrhythmia, nausea and vomiting, and psychic symptoms. Stable hemodynamic parameters without significant respiration depression or other adverse events were found with the combination of propofol and esketamine. Eberl et al. [16] also found no differences in the incidence of adverse events with low-dose esketamine combined with propofol compared with alfentanil combined with propofol for ERCP. Yang’s study did not find significant clinical complications and psychotomimetic effects of the coadministration of a relatively high dose of esketamine and propofol for the procedure of gastrointestinal endoscopy [15].

In our study, psychic symptoms such as involuntary tremors, involuntary lacrimation, agitation, and nightmares were observed, but no hallucinations, delirium, or depression were found. Previous studies have also shown that the combination of ketamine and propofol is beneficial for reducing the incidence of psychotomimetic effects [11, 18, 19]. However, the mechanisms underlying this disorder remain unknown. MMSE is a relatively simple scale of cognitive function and it is widely used in the diagnosis of psychopathology-related disorders [20]. In one study, MMSE scores measured 15 min after low-dose ketamine combined with propofol was applied to patients undergoing ambulatory surgery were higher than those in the propofol alone group in three domains of cognitive function in MMSE (orientation, attention, and recall) [21]. However, we did not find any differences in MMSE scores among the four groups in our study. To conclude, esketamine is effective for GI endoscopy sedation with proper safety.

There are still some limitations to our study. First, since ketamine was not available in most hospitals in China, we could not set up a group of ketamine patients for comparison with esketamine. Second, this study did not apply the depth of anesthesia to assess the depth of sedation, but we judged the depth of sedation through the disappearance of eyelash reflex and the absence of cough and body movement. Third, the doses of esketamine set in this study were all subanesthetic doses, and the doses used were relatively low. Additional studies with 0.3 mg/kg, 0.4 mg/kg, and 0.5 mg/kg esketamine can be considered. Fourth, since we assessed the awakening status of patients at 2-min intervals, the recording of awakening time may be delayed, so we adopted the method of awakening status assessment (more than 6 min was defined as delayed awakening). Finally, multicenter studies with larger sample sizes are needed to confirm our findings.

## Conclusions

In this study, we observed that in painless gastrointestinal endoscopy, propofol combined with 0.2 mg/kg esketamine had a shorter induction time, lower incidence of cough and body movement, and less propofol consumption per minute than propofol alone; there was no effect on recovery time, hemodynamic stability, postoperative cognitive function, and the incidence of adverse events and psychotomimetic effects.

## Data Availability

The raw data are available from the corresponding author on reasonable request.
